# Conjugation of Functionalized SPIONs with Transferrin for Targeting and Imaging Brain Glial Tumors in Rat Model

**DOI:** 10.1371/journal.pone.0037376

**Published:** 2012-05-17

**Authors:** Weili Jiang, Hui Xie, Devina Ghoorah, Yalei Shang, Haojun Shi, Fang Liu, Xiangliang Yang, Haibo Xu

**Affiliations:** 1 Radiology Department of Union Hospital, Huazhong University of Science and Technology, Wuhan, Hubei, People's Republic of China; 2 Department of Information Processing, China Patent Information Center, Wuhan, People's Republic of China; 3 College of Life Science and Technology, Huazhong University of Science and Technology, Wuhan, Hubei, People's Republic of China; National Institute of Health, United States of America

## Abstract

Currently, effective and specific diagnostic imaging of brain glioma is a major challenge. Nanomedicine plays an essential role by delivering the contrast agent in a targeted manner to specific tumor cells, leading to improvement in accurate diagnosis by good visualization and specific demonstration of tumor cells. This study investigated the preparation and characterization of a targeted MR contrast agent, transferrin-conjugated superparamagnetic iron oxide nanoparticles (Tf-SPIONs), for brain glioma detection. MR imaging showed the obvious contrast change of brain glioma before and after administration of Tf-SPIONs in C6 glioma rat model in vivo on T2 weighted imaging. Significant contrast enhancement of brain glioma could still be clearly seen even 48 h post injection, due to the retention of Tf-SPIONs in cytoplasm of tumor cells which was proved by Prussian blue staining. Thus, these results suggest that Tf-SPIONs could be a potential targeting MR contrast agent for the brain glioma.

## Introduction

Magnetic resonance imaging(MRI) is currently the preferred method in clinical diagnosis of brain glioma, with the characteristics of non-invasive, high spatial resolution, multi-direction, multi-sequence and multi-parameter imaging capability [1]. Current approach for diagnosis and grading of brain glioma is utilizing MR imaging with administration of gadolinium chelate-based contrast agents [2]. However, gadolinium chelate-based contrast agents suffer issues of short imaging time, fast diffusion, and certain side effects in patients, especially of nonspecifically targeting brain glioma [3], [4]. Developing a novel contrast agent which can overcome these limitations has currently become a challenge. Superparamagnetic iron oxide nanoparticles (SPIONs) have recently emerged as magnetic nanocrystals which have been explored preclinically for their ability to aid in brain tumor visualization on MRI [5], [6]. SPIONs not only have high relaxation rate but also display outstanding biocompatibility, biodegradability and nontoxicity [7]. SPIONs, however, have no specificity to target brain glioma [8]. Nanoparticles' surface modified with specific ligands, can improve the contrast enhancement effect of tissue on MRI [9], [10]. Furthermore, till now, there are only limited examples of SPIONs successful for glioma on MRI [11], [12]. Since targeting ligands plays a crucial role in detection of brain tumor, developing a specific MRI contrast agent conjugated with a target-specific ligand for brain glioma would add to the current avenue for glioma diagnosis.

Transferrin (Tf) is a single-chain glycoprotein containing about 700 amino acids, and belongs to the transferrin family [13]. Tf is one of the most widely used tumor targeting ligand since tumor cells express Tf receptors (TfRs) more commonly than other general cells [14]. Not only does Tf bind to TfRs with high affinity but the receptors are found to be overexpressed in several human carcinomas including breast, ovary, and brain cancers such as glioma and glioblastomas [15], [16], [17]. These findings make the transferrin family potentially valuable as cancer biomarkers. Besides, transferrin receptor-mediated endocytosis of transferrin-bound iron complexes has been stated as one of the most characteristic processes in cell biology [13], [18]. Furthermore, owing to the TfRs existing on the blood-brain barrier (BBB), conjugation of transferrin with a contrast agent, e.g. SPIONS, may allow Tf to circumvent the impermeable barrier, be readily delivered to brain, and concomitantly enhancing their role as a carrier [19], [20]. In view of developing a multifunctional contrast agent, TfRs deserve particular mention for embodying an important target for glioma therapy, as they have the ability to downregulate neural tumor cell cycles and glioma expansion [16]. Thus, all these factors indicate that Tf conjugated SPIONs (Tf-SPIONs) could be potentially investigated as specific contrast agents in targeting brain glioma. Although lactoferrin(Lf) which belongs to the transferrin family, offers a useful new tool as a brain glioma biomarker, it has not been widely used or translated for use in medical imaging, and part of the reason being the lack of lactoferrin receptors monoclonal antibodies. Thus, the probes (Tf-SPIONs) represent potentially valuable new imaging tools for application to a number of clinically relevant diseases that involve Tf receptors overexpression.

In this study, we aim to determine: firstly, the development and characterization of Tf-SPIONs as well as whether it is suitable for MR imaging; secondly, whether Tf-SPIONs could be specifically and efficiently internalized by C6 glioma cells and detected by MR imaging; and thirdly, whether Tf-SPIONs could act as a specific targeting MRI contrast agent for brain glioma in vivo without saturation of the TfRs on BBB.

## Results

### Characterization of Tf-SPIONs

**Figure 1:**
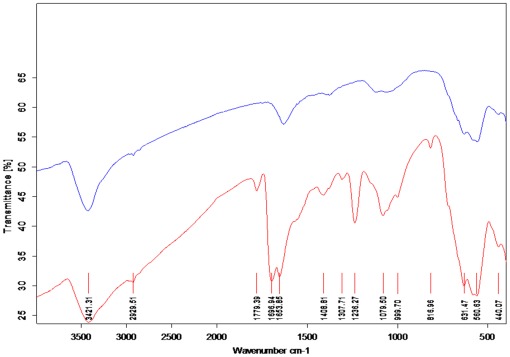
Fourier transform infrared spectroscopy spectra of SPIONs (blue curve) and Tf-SPIONs (red curve), respectively.

SPIONs surface chemistry and Tf conjugation were confirmed by FTIR. Fig. 1 shows the FTIR spectra of SPIONs (blue curve) and Tf-SPIONs (red curve). The results showed that peaks at about 630, 590 and 450 cm^−1^ both from SPIONs (blue curve) and Tf-SPIONs (red curve) were typical Fe-O absorption bands. After conjugation with Tf, amide I band peak around 1696 cm^−1^ and amide II band peak around 1653 cm^−1^ were observed, as represented by the red curve of Fig. 1.

**Figure 2:**
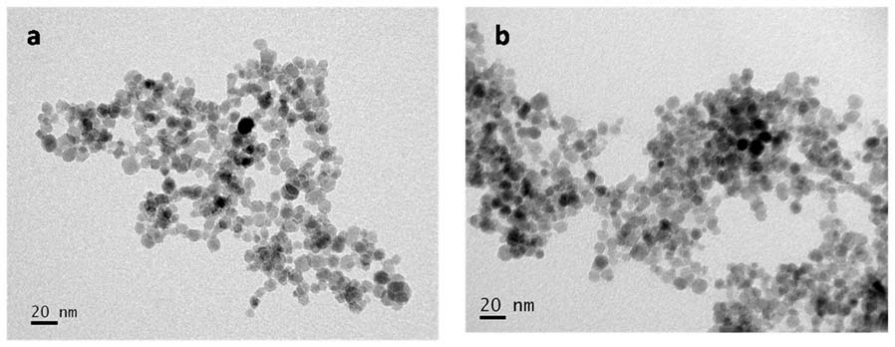
TEM image of SPIONs (**Fig. 2a**) and Tf-SPIONs (**Fig. 2b**), respectively.

The morphology and size distribution of SPIONs and Tf-SPIONs by TEM images were shown in Fig. 2. The morphology of Tf-SPIONs was similar to SPIONs. The average particle size calculated from the statistical analysis of SPIONs was 9.3±0.5 nm, and Tf-SPIONs average particle size was 14.1±0.6 nm.

**Figure 3:**
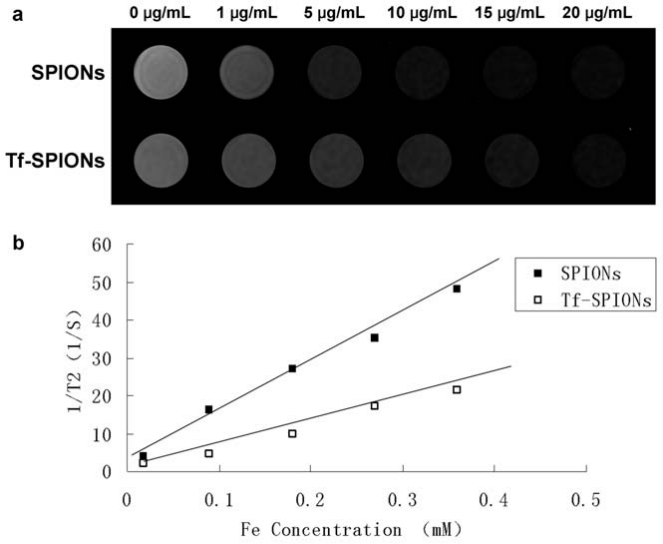
Magnetic properties of nanoparticles. (a) MR T2-weighted images of SPIONs and Tf-SPIONs; (b) T2 relaxation rate (1/T2, S^−1^) of SPIONs and Tf-SPIONs.

The T2 relaxation time was the linear fit with the particle concentration. When the Tf-SPIONs concentration increased, the signal intensity decreased (Fig. 3a). The T2 relaxivity coefficient value (1/T2, S^−1^) of Tf-SPIONs from the MRI was 64.3 S^−1^ mM^−1^ (Fig. 3b). This is suggestive of their prospective use as a negative MRI contrast agent in T2-weighted imaging.

### In vitro Study

**Figure 4:**
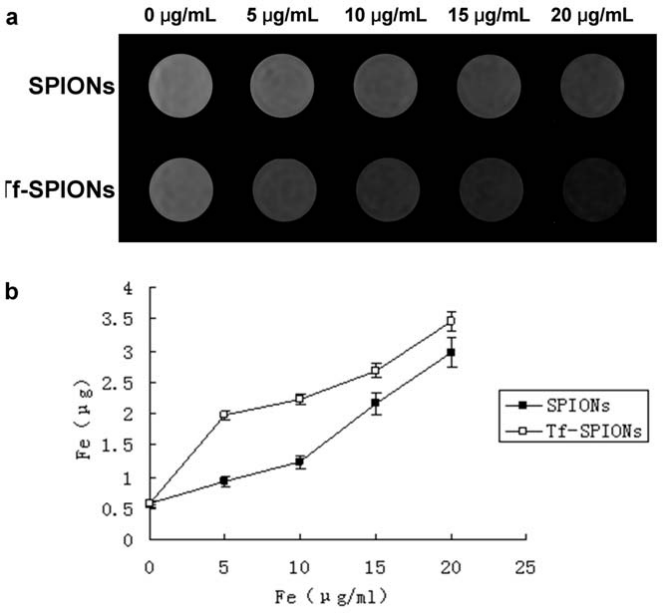
Iron uptake in C6 cells treated with nanoparticles. (a) In vitro T2-weighted MR. imaging of C6 cells with Tf-SPIONs. Under 0 µg/ml column: C6 cells only without SPIONs or Tf-SPIONs; SPIONs row: C6 cells with SPIONs; Tf-SPIONs row: C6 cells with Tf-SPIONs. (b) Uptake curves of C6 cells incubated with SPIONs or Tf-SPIONs.

MR T2-weighted images of the nanoparticles cultured with C6 cells of various concentrations are shown in Fig. 4a. Cells treated with Tf-SPIONs showed a significant negative contrast enhancement at each of the nanoparticle concentrations, compared to the tumor cells incubated with bare SPIONs. The signal enhancement displayed in the cells cultured with Tf-SPIONs is consistent with the cellular uptake studies. The iron absorbed by C6 cells incubated with Tf-SPIONs was much higher than cells incubated with SPIONs at different nanoparticle concentrations (Fig. 4b). This result demonstrated that, C6 cells could efficiently and specifically internalize Tf-SPIONs compared to SPIONs.

**Figure 5:**
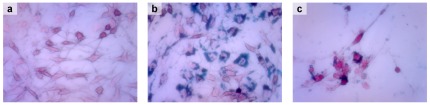
C6 cells were stained with Prussian blue. (a):C6 cells only; (b):C6 cells incubated with Tf-SPIONs ; (c):C6 cells incubated with SPIONs.

After Prussian blue staining, Tf-SPIONs and SPIONs were both found in the cytoplasm of C6 cells and appeared as blue spots. C6 cells incubated with Tf-SPIONs (Figure 5b) contained large amount of blue spots in the cytoplasm, whereas only a few blue particles were observed in the cytoplasm of C6 cells cultured with SPIONs (Figure 5c).

### In vivo Study

**Figure 6:**
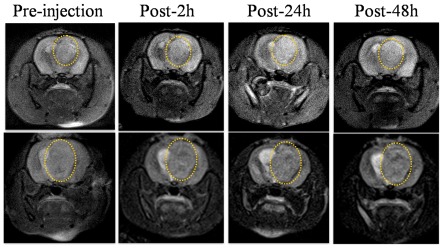
In vivo T2-weighted MR images of rats' brain bearing C6 gliomas. Upper row: acquired after administration of SPIONs (12 mg/kg); lower row: acquired after administration of Tf-SPIONs (12 mg/kg). The tumor was marked by yellow circle. After injection of Tf-SPIONs, the changing area of decreased MR signal intensity increased gradually with timing up to 48 h in comparison to the preinjection image of tumor.

To verify whether Tf-SPIONs could act as a specific targeting MRI contrast agent for brain tumor, we performed MR imaging with male Wistar rat brain glioma models (n = 16) in vivo and compared with the resulting findings following administration of Tf-SPIONs and SPIONs. Fig. 6 showed the MR imaging of rat brain tumor, after intravenous injection with SPIONs or Tf-SPIONs as a contrast agent at the dose of 12 mg Fe/kg. The T2-weighted images of rat brain injected with Tf-SPIONs obtained 2 h, 24 h and 48 h post-injection exhibited a region of apparent hypointensity compared to the baseline on T2-weighted images (Fig. 6). The hypointense region within the glioma lesion is indicative of nanoparticle accumulation, which causes reduction in signal intensity on T2-weighted images. After injection of Tf-SPIONs, the MR signal intensity at the tumor site decreased by 32%–65% in comparison to the preinjection image of tumor, and the area of signal change of the tumor increased gradually until 48 h (Fig. 6). Conversely, the post-injection images of rat administrated with SPIONs showed almost no signal reduction within the tumor tissue.

**Figure 7:**
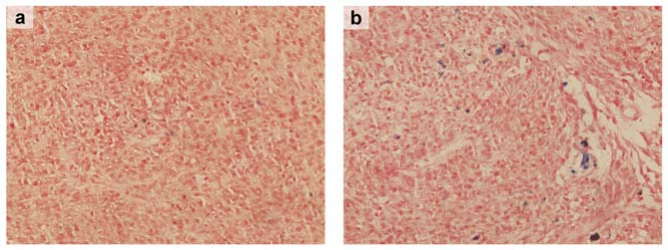
Histochemical analysis of the tumor tissue. Rats were sacrificed after 48 h of injection of nanoparticles. (a): sections were stained with Prussian blue with SPIONs (400×); (b):with Tf-SPIONs (400×).

Finally, to confirm that the signal intensity change of tumor following administration of Tf-SPIONs was due to the accumulation of Tf-SPIONs in the tumor tissue, rather than gadolinium chelate-based contrast agents or SPIONs which were trapped in the tumor vascularity, we performed the Prussian blue staining of the tumor tissue species after the MR imaging. Prussian blue staining demonstrated the accumulation of Tf-SPIONs in the tumor tissue up to 48 h after the injection (Fig. 7). Tf-SPIONs were found to be located in the cytoplasm of the tumor cells, and consequently this accumulation manifested as reduction in signal intensity on T2-weighted images of brain glioma as shown in Fig. 6. Concurrently, Prussian blue staining proved negative in tumor-bearing rats with SPIONs.

## Discussion

In this study, three main findings were generated: first, Tf-conjugated SPIO (Tf-SPIONs) is suitable for MR imaging; secondly Tf-SPIONs could be specifically and efficiently internalized by C6 glioma cells, along with prolonged retention and detection by MR imaging; and thirdly Tf-SPIONs could create the obvious contrast change of brain glioma in vivo following administration of Tf-SPIONs owing to the larger accumulation of Tf-SPIONs in the cytoplasm of brain glioma. Thus, the results suggested that Tf-SPIONs could act as potential and specific MRI targeting contrast agent for brain glioma, while possessing the characteristics of long retention in glioma and higher T2 relaxation rate.

Characterization of Tf-SPIONs in physics is suitable for an MRI contrast agent. Tf-SPIONs average particle size was 14.1±0.6 nm. Their relatively small size and fine dispersion provide favorable in vivo pharmacodynamic properties and cellular uptake. R2, the relaxation rate, reflects the degree of the MRI contrast agent on T2 relaxation rate. When the paramagnetic nanoparticle T2 relaxation rate is higher, its ability to shorten proton relaxation time is stronger and the MRI signal contrast between tissues becomes more obvious. Hence, this kind of nanoparticle is more suitable for use as negative MRI contrast agent [21]. The MRI results indicated that the T2 relaxation rate of Tf-SPIONs was 64.3 S^−1^ mM^−1^,thus being higher than that of MRI contrast agent Combidex ® (53.1 S^−1^ mM^−1^), which was used in clinical studies for the detection of lymph node metastasis [22]. Therefore, these results indicated that Tf-SPIONs with its higher T2 relaxation rate, could be used as MRI contrast agent.

Previous studies demonstrated that SPIONs absorbed by glioma cells in vitro is concentration dependent [23], and our study corresponded to those observations. As shown in Figure 4a, C6 cells incubated with various concentrations of SPIONs or Tf-SPIONs could induce signal intensity decrease. With increasing concentrations, the signal intensity decreased more obviously. Moreover, our study revealed that Tf-SPIONs incubated cells displayed significantly negative contrast enhancement on T2-weighted imaging at the respective nanoparticle concentrations, which was in contrast to the cells incubated with SPIONs.

The intracellular uptake of nanoparticles results are consistent with the signal enhancement observed in the cells incubated with the nanoparticles on MRI. Larger degree of internalization of Tf-SPIONs by C6 cells rather than that of SPIONs, suggested that the increase of cellular uptake was most probably due to the conjugation with Tf. Prussian blue staining provided further evidence that large quantity of Tf-SPIONs accumulated in the cytoplasm of C6 cells as shown in Figure 5; whereas relatively smaller amounts of SPIONs were observed. These findings demonstrate that Tf-SPION has high specificity for C6 glioma cells, as well as high efficiency.

A 3.0 T MR scanner was utilized, to analyse the ability of Tf-SPION to act as a contrast agent for brain glioma in vivo and without saturation of the TfRs on BBB. As shown in Figure 6, significant enhancement of brain glioma was observed with Tf-SPIONs accumulating in tumor tissues, and possessing reasonable signal level, as well as long retention in tumor center up to 48 h post-injection. On the other hand, we observed low tumor uptake and not much contrast change of the tumor before and after administration of SPIONs. The result of Prussian blue staining demonstrated further that Tf-SPIONs could get specifically assembled into tumor tissues in vivo, which was consistent with the MR imaging. As shown in Figure 7, Tf-SPIONs accumulating in glioma was significantly higher than that of SPIONs. As for SPIONs, the majority of nanopartilces were probably trapped by the basement membrane and only a few of them could leak into the periphery of glioma area by the increased vascular permeability [12], [23]. In addition, Muldoon et al suggested that Feridex may remain in the basement membrane rather than pass through incompetent BBB into the brain [24]. In our study, no significant signal change was observed in tumor after injection of SPIONs probably due to most of them being trapped by the basement membrane. Recently, Gao HL et al compared the brain uptake of Tf-conjugated polymersomes with that of lactoferrin [25]. The results showed that Tf was more effective than lactoferrin in brain targeting. This suggests that TfR on the BBB may be involved in Tf transport across the BBB. Therefore, through receptor-mediated endocytosis, Tf could cross the BBB and undergo uptake by tumor cells.

In our previous study, the Lf receptor was expressed on the glioma [12], but to the best of our knowledge, the Lf receptor monoclonal antibody has not yet been found. In addtion, although the Tf receptor monoclonal antibody (OX26) has good specificity, the expensive price and the difficulty in preparation, limit the further application of OX26. Alternatively, Tf is cheap and is easy to prepare, and thereby Tf stands great potential for the clinical application.

The biocompatibility of SPIONs is one of the most important concerns in vivo application. Bourrinet et al. found that no treatment-related clinical signs were observed for rats injected with 2, 13 or 40 mg/kg of Ferumoxtran-10, while rats receiving 126 and 400 mg/kg showed clinical symptoms associated with toxicity [26]. Chertok et al. used 12 mg/kg of SPIONs as a drug carrier for the treatment of rat glioma, the result of which showed that no toxic effects were observed on the body [27]. In our study, we used 12 mg/kg of Tf-SPIONs, which was far less than the dose reporting toxic reactions in rats. SPIONs conjugated with the targeting ligand demonstrated the ability to be selectively uptaken by target tissue and accordingly reduce the toxicity to other tissues and organs. At present, improving site specific distribution, while at the same time reducing spread to other tissues is the keypoint in brain drug delivery sytem [25]. Considering this fact, it is noteworthy that Tf-SPIONs utilized in imaging for glioma in rats basically do not produce toxic effects, nevertheless their tissue distribution requires further study.

Although the probes reported here support the potential use of Tf-SPIONs for brain glioma imaging, significant work is required to improve the probes. For instance, improvement of the probes for the detection of the peripheral tumor or tumor cells within the surrounding edema. The work presented here with Tf-SPIONs serves as the foundation for future studies of targeted glioma imaging and broad applications of MR imaging other tumors with overexpressed Tf receptors as well as assessment of the recurrent tumor after tumor resection and postoperative radiotherapy.

## Materials and Methods

### Synthesis and Characterization of Tf-SPIONs

Transferrin (Tf) from human transferrin was purchased from Sigma-Aldrich (St. Louis, Mo, USA). Rats (male Wistar, 250–300 g) were provided by Hubei center for Disease Control and Prevention.

Tf-SPIONs was synthesized through EDC conjugation, following the method in our previous report [12].

The formation of Tf-SPIONs was verified by fourier transform infrared spectroscopy (FTIR, VERTEX 70, Bruker, Ettlingen, Germany). For each sample, 2 mg of dried nanoparticles was mixed with 200 mg of KBr and pressed into a pellet for analysis.

The morphology and size distribution of nanoparticles were examined by transmission electron microscopy (TEM, JEM-2010, JEOL, Tokyo, Japan) at 200 kV. The particle size was quantified through a visual analysis of -200 particles in the TEM micrographs.

The MR T2 relaxivity of the nanoparticles were determined using 3.0-T whole body MR scanner (MAGNETOM Trio, A Tim System 3T, Siemens, Munich, Germany) in combination with 8-channel wrist joint coil. The particles were diluted in distilled water, with iron concentrations in the range of 0–20 µg/mL. Samples were transferred to a 96-well plate. T2-weighted images were acquired using a multi-slice spin echo sequence. The parameters were set as follows: field of view (FOV) 120 mm, base resolution, 384×384, slice thickness 1.5 mm, multiple echo times (TE) 20, 40, 60, 80,100,120,140 ms, repetition time (TR) 2000 ms, and scan time was 13–14 min. T2 relaxation rates were plotted against the iron concentrations in the particle dilutions. The relaxivity was determined by a linear fit.

### In vitro Study

C6 rat glioma cells were provided by the Institute of Biochemistry and Cell Biology of Tongji Medical School (Wuhan, China) [28]. Cells were cultured in Dulbecco modified Eagle's medium (DMEM) supplemented with 10% fetal calf serum (Gibco, USA) and antibiotics penicillin (100 IU/mL)/streptomycin (100 mg/mL) in a humidified atmosphere with 5% CO2 at 37°C.

C6 Cells were incubated with SPIONs and Tf-SPIONs with different iron concentrations (5, 10, 15 and 20 mg/mL) at 37°C for 0.5 h. The cells were washed and then suspended by 300 mL 0.5% agarose gel. Samples were then quickly transferred to a 96-well plate. MR imaging was performed on a 3.0-T magnet (MAGNETOM Trio, A Tim System 3T, Siemens, Munich, Germany). T2-weighted spin echo multi-slice pulse sequence was used. The parameters were as following: field of view (FOV) 120 mm, base resolution, 384×384, slice thickness 1.5 mm, multiple echo times (TE) 20, 40, 60, 80,100,120,140 ms, repetition time (TR) 2000 ms, and scan time was 13–14 min.

C6 Cells were cultured with various nanoparticles at different iron concentrations in the range of 0–20 mg/mL for 0.5 h at 37°C. The cells were washed and collected. Intracellular iron content was determined by fast sequential atomic absorption spectrometer (Spectr AA240FS, Varian, Palo Alto, USA). Cells were added to 0.5 mL of perchloric acid and 2 mL nitric acid. The mixtures were heated for 1 h and then cooled down to room temperature. 2 mL distilled water was used to wash inner wall of a conical flask and was then transferred to a centrifuge tube for measurement. The parameter of Spectr AA240FS is following: Wavelength, 248.3 nm; Slit, 0.2 nm; Lamp current, 5 mA; Maximum Ash, 800°C; Atomize, 2300°C; Characteristic Mass, 2.

Untreated cells were seeded onto 6-well plates at a density of 2×10^6^ cells/well. 24 h following seeding, C6 glioma cells were incubated with Tf-SPIONs and SPIONs at a concentration of 20 µg/mL for 2 h. C6 glioma cells were treated with PBS as the control. After labeling, the samples were washed thrice with cell culture medium and thrice with PBS. The cells were soaked with 4% paraform and washed with PBS, followed by staining with Prussian Blue solution (equal volume of 6% hydrochloric acid and 2% potassium ferrocyanide) for 30 min. Afterwards, the treated cells were counterstained with Nuclear Fast Red solution for 5 min. The samples were then examined and mounted under optical microscope. The percentage of Prussian blue stained-positive cells was counted under a random field of view (200×) and the procedure was repeated 10 times for each sample.

### In vivo Study

All animal experiments were approved by the Institutional Animal Care and Use Committee at Union Hospital in the Huazhong University of Science and Technology. Sixteen male wistar rats were anesthetized with 10% Chloral Hydrate (i.p., 100 g/0.4 mL) and immobilized in a stereotactic frame. A 1-mm diameter burr hole was drilled through the skull 1 mm posterior and 3.0 mm lateral to the bregma. A 20 µl micro- injector was then used to inject 10 µl of the C6 cell suspension (1×10^6^ C6 cells in DMEM). The injection was done slowly over 10 min, maintained for 10 min and the needle was withdrawn after another 10 min. The burr hole was filled with bone wax to prevent extracerebral extension of the tumor and the skin was closed with nonmagnetic sutures. MRI was performed on eight tumor bearing rats administered with SPIONs via their tail veins, and on the other eight tumor bearing rats which were injected with Tf-SPIONs via the same route.

Rats were injected with 12 mg/kg body weight nanoparticles via tail vein, following anesthesia. 8-channel wrist joint coil was used for MRI after the rats were placed in the imaging chamber. The time course of nanoparticle distribution in the rat brain was monitored by T2-weighted spin echo sequence. The parameter were as following: field of view (FOV) 80 mm, base resolution, 192×192, slice thickness 1 mm, echo time (TE) 91 ms, repetition time (TR) 6540 ms. The signal intensity of tumor was obtained at the region of interest (ROI) of 2 mm^2^, placed at the tumor site in the same slice on T2WI, before and after the administration of contrast agent such as Tf-SPIONs or SPIONs. The signal change of the tumor was calculated using the following formula: (SIpre-SIpost)/SIpre×100%, where SIpre and SIpost were the signal intensity of tumor before and after the administration of contrast agent.

Histological analysis of brain tissues was performed two days post-injection. Rats were perfused with 250 mL Sodium Chloride and 250 mL 4% paraform, brain tissues were removed and fixed in 4% paraform for at least 24 h. The paraform fixed sections were then embedded in paraffin and sectioned. Sections were subsequently stained with H&E and Prussian Blue per standard clinical laboratory protocol.

### Statistical analysis

The data were presented as mean ± SE.
